# DARS expression in JAK2V617F-positive myeloproliferative neoplasms: immunohistochemical analysis and clinical associations

**DOI:** 10.1007/s00277-026-06934-0

**Published:** 2026-03-27

**Authors:** Aya Mohamed Adel Arafat, Heba Adel AbdEl Ghaffar, Ahmed M. Khallaf, Sufyan Ahmed Mokhtar Younes, Mohamed Abdelkader Morad, Shirihan Mahmoud Anwar Mahgoub

**Affiliations:** 1 Department of Clinical and Chemical Pathology, Faculty of Medicine, Cairo University, Cairo, Egypt; 2 Department of Internal Medicine and Clinical Hematology, Faculty of Medicine, Beni-Suef University, Beni-Suef, Egypt; 3 Department of Pathology, Faculty of Medicine, Alasmarya Islamic University, Zliten, Libya; 4 Department of Internal Medicine, Clinical Hematology Unit, Kasr Al Ainy Faculty of Medicine, Cairo University, Cairo, Egypt

**Keywords:** DARS, Myeloproliferative neoplasms, JAK2V617F, Aspartyl-tRNA synthetase, Immunohistochemistry

## Abstract

Aspartyl-tRNA synthetase (DARS) is implicated in several cancers, but its role in BCR::ABL-negative JAK2V617F-positive myeloproliferative neoplasms (MPNs) is unclear. This study evaluated DARS expression in MPN subtypes and its associations with clinical parameters and survival. Diagnostic bone marrow biopsies from 121 JAK2V617F-positive MPN patients (PV, n = 34; ET, n = 25; PMF, n = 54; MPN-U, n = 8) were stained on a Ventana BenchMark XT immunostainer using anti-DARS antibody (ABclonal A6574). DARS immunoreactive score (IRS) was determined by multiplying intensity (0–3) and percentage of positive cells (0–4). Inter-observer agreement was excellent (κ = 0.89, 95% CI: 0.83–0.95). Patients were stratified using the cohort’s median cutoff (IRS = 9) for survival analysis. Statistical tests included Kruskal-Wallis with effect sizes and multivariate Cox regression. DARS IRS differed across subtypes (H = 14.19, p = 0.003, η²=0.10): PV median 9 with IQR (9–12), ET 9 (8–12), PMF 8 (6–9), and MPN-U 10.5 (6–12). PV vs. PMF differences: intensity p = 0.001 (r = 0.35), IRS p < 0.001 (r = 0.38). DARS IRS correlated inversely with spleen size (ρ=–0.266, p = 0.003, 95% CI: − 0.43 to − 0.09), LDH (ρ=–0.194, p = 0.033), and fibrosis grade (ρ=–0.280, p = 0.002), and positively with hemoglobin (ρ = 0.308, p = 0.001, 95% CI: 0.13–0.47). High DARS expression independently predicted improved leukemia-free survival (LFS) (HR = 0.42, p = 0.007, 95% CI: 0.22–0.80) after adjusting for age, subtype, and fibrosis. DARS is differentially expressed in MPN subtypes. Its association with enhanced LFS identifies DARS as a potential prognostic biomarker warranting validation in larger cohorts.

**Supplementary Information:**

The online version contains supplementary material available at 10.1007/s00277-026-06934-0.

## Introduction

BCR::ABL1-negative myeloproliferative neoplasms (MPNs) represent a group of clonal hematopoietic disorders characterized by excessive proliferation of mature blood cells in the bone marrow. The major subtypes include polycythemia vera (PV), essential thrombocythemia (ET), and primary myelofibrosis (PMF), with the JAK2V617F mutation being present in approximately 95% of PV patients and 50–60% of ET and PMF patients [1, 2]. These disorders share common pathophysiological mechanisms involving dysregulated JAK-STAT signaling pathways but exhibit distinct clinical presentations, complications, and prognoses [3].

Aspartyl-tRNA synthetase (DARS), encoded by the DARS gene located on chromosome 2q21.3, is an essential enzyme responsible for charging tRNA with aspartic acid during protein synthesis [4]. Beyond its canonical role in translation, DARS has emerged as a multifunctional protein with roles in cell signaling, immune responses, and tumorigenesis [5, 6]. Recent studies have implicated DARS in various malignancies, including melanoma, glioblastoma, colon cancer, and gastric cancer, where its overexpression is associated with tumor progression, metastasis, and poor prognosis [7–16]. The landmark study by Xiong et al. demonstrated a significant DARS overexpression in BCR::ABL1-negative MPNs by immunohistochemistry [17]. However, this study included a limited number of cases and lacked a comprehensive statistical analysis of differential expression patterns across subtypes or detailed correlations with clinical parameters. Furthermore, recent advances in understanding aminoacyl-tRNA synthetases in cancer biology have revealed their potential as therapeutic targets and prognostic biomarkers [18, 19]. The heterogeneity observed within MPNs, particularly in PMF, in which patients can present with prefibrotic or fibrotic phases, necessitates the identification of putative biomarkers to improve disease classification and risk stratification [20, 21]. Additionally, understanding molecular mechanisms underlying MPN pathogenesis beyond JAK2V617F mutation is crucial for developing targeted therapeutic strategies [22]. This study aims to provide a comprehensive analysis of DARS expression in a larger cohort of BCR::ABL1-negative JAK2V617F-positive MPN patients, with detailed statistical evaluation of expression differences across subtypes, systematic investigation of correlations with clinical parameters, and survival outcome analysis. We hypothesize that DARS expression patterns correlate with specific MPN subtypes and clinical characteristics, potentially serving as a valuable biomarker for disease classification and prognosis.

## Materials and methods

### Study design and Patients

This retrospective cohort study included 121 patients with BCR::ABL1-negative, JAK2V617F-positive MPNs (confirmed by allele-specific qPCR) to ensure biological homogeneity in JAK-STAT signaling and enable robust subtype comparisons (> 80% power). CALR/MPL-mutated cases were excluded as they confer distinct phenotypes that could confound DARS expression analyses, following precedents in driver-stratified MPN biomarker research. The diagnostic bone marrow biopsies (BMB) were from treatment-naïve MPNs. All diagnoses met the 2021 World Health Organization (WHO) criteria [23] and were confirmed by blinded biopsy review. The cohort comprised 34 PV patients, 25 ET patients, 54 PMF patients (16 prefibrotic and 38 fibrotic), and 8 MPN-U patients. All patients were diagnosed and treated at Cairo University Hospital, Beni-Suef University Hospital, and Alasmarya Islamic University Hospital between 2018 and 2023.

### Sample size calculation

Sample size calculation was performed using G*Power 3.1.9.7 software. To detect a medium effect size (Cohen’s d = 0.5) with 80% power at α = 0.05 for comparing DARS expression across four MPN subtypes, a minimum sample size of 108 patients was required. Our study included 121 patients, providing adequate power (84%) for the primary analysis.

### Ethics statement

This study was reviewed and approved by the Beni-Suef University Ethics Committee (approval number: FMBSUREC/01072025/Khalaf). Written informed consent was obtained from all participants before enrolment. All procedures involving human participants were performed in accordance with the ethical standards of the institutional research committees and with the 1964 Helsinki Declaration and its later amendments.

### Inclusion and exclusion criteria

Inclusion criteria: (1) Age ≥ 18 years; (2) Confirmed diagnosis of BCR::ABL1-negative MPN according to WHO 2021 criteria [23]; (3) Positive JAK2V617F mutation confirmed by allele-specific PCR; (4) Availability of BMB specimens suitable for immunohistochemical analysis; (5) Complete clinical and laboratory data available for analysis.

Exclusion criteria: (1) Secondary polycythemia or thrombocytosis; (3) Prior treatment with JAK inhibitors at the time of biopsy; (4) Insufficient tissue samples for analysis; (5) Concomitant hematological malignancies.

### Specimen processing and immunohistochemistry

Formalin-fixed, paraffin-embedded BMB specimens were sectioned at 4 μm thickness and processed for immunohistochemical staining. DARS expression was evaluated using a validated anti-DARS antibody (Catalog No: A6574, ABclonal, 500 W Cummings Park, Ste. 6500 Woburn, MA 01801, RRID: AB_2767168) at a 1:50 dilution. Antibody validation was performed using positive controls (normal breast tissue known to express DARS) and negative controls (omission of primary antibody) [24].

Immunohistochemistry was performed following standard protocols using an automated immunostainer (Ventana BenchMark XT, Roche Diagnostics). Antigen retrieval was carried out with a cell conditioning 1 buffer (CC1 buffer) for 60 min, followed by incubation with the primary antibody for 60 min. A rabbit linker was applied for 10 min, after which the sections were treated with horseradish peroxidase (HRP) for 13 min. Visualization was achieved using 3,3′-diaminobenzidine (DAB) for 5 min, and counterstaining was performed with hematoxylin for 3 min.

Quality control measures included daily calibration of the automated stainer, use of positive and negative controls with each run, and batch processing to minimize inter-batch variation.

### DARS expression scoring

**Fig. 1:**
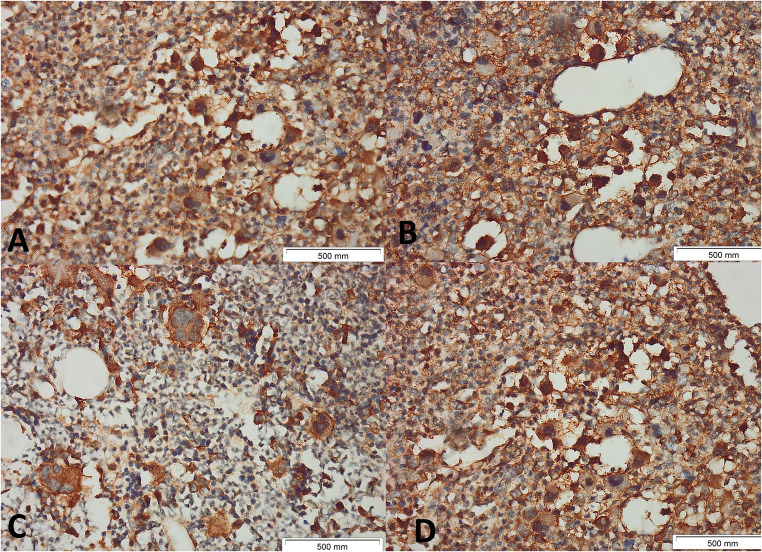
Immunohistochemical Expression of DARS in Bone Marrow Biopsies from Patients with JAK2V617F-positive Polycythemia Vera. Representative immunohistochemical staining for aspartyl-tRNA synthetase (DARS) expression in bone marrow biopsies (BMB) from patients with polycythemia vera (PV). Panels A–B show intense (+ 3) cytoplasmic and membranous DARS positivity in megakaryocytes and varying degrees of positivity in megakaryocytes and myeloid precursors at 400× magnification. Panels C-D show moderate (+ 2) cytoplasmic and membranous DARS positivity in megakaryocytes and varying degrees of positivity in megakaryocytes and myeloid precursors at 400× magnification. Scale bars represent 500 μm in each image

**Fig. 2:**
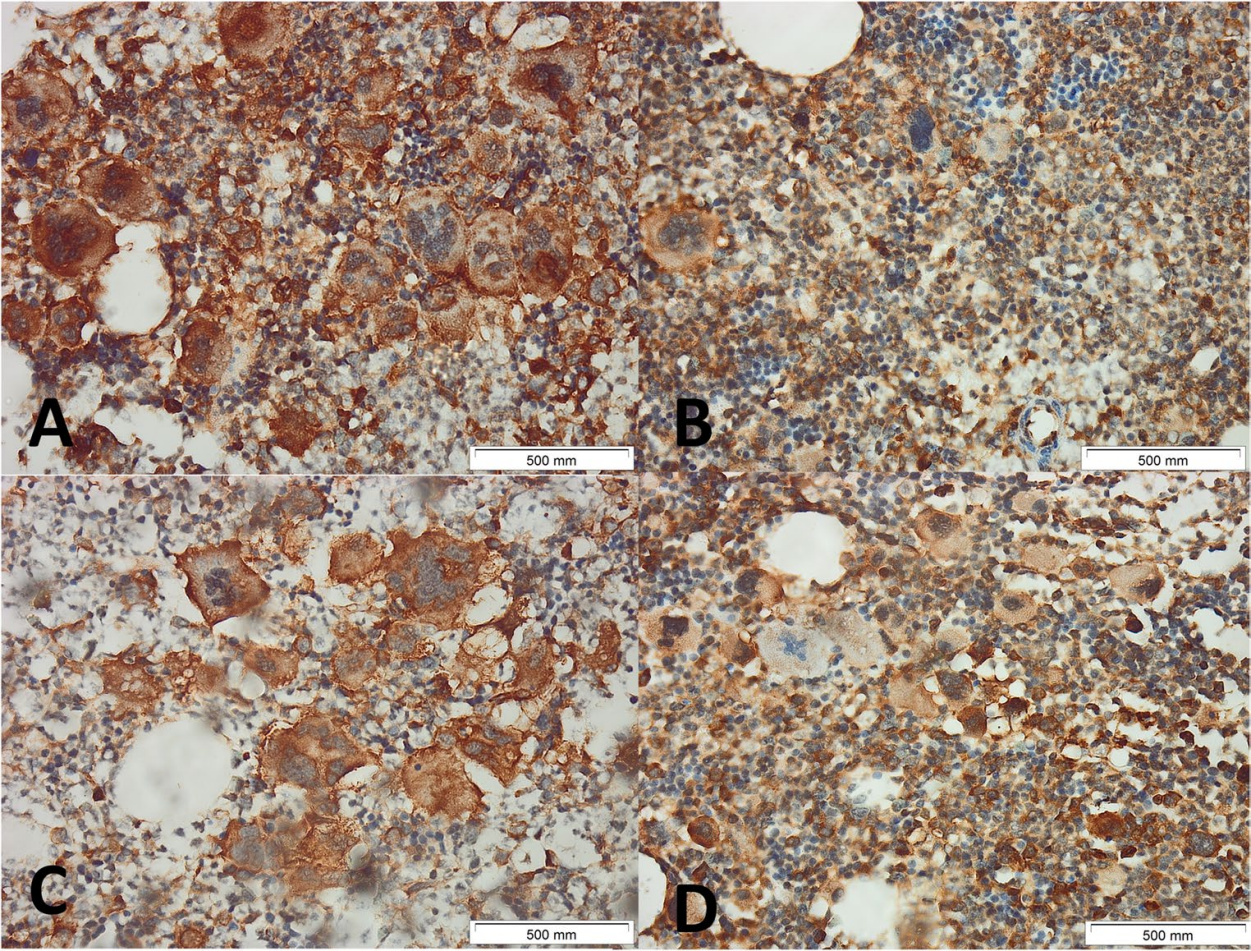
Immunohistochemical Expression of DARS in Bone Marrow Biopsies from Patients with JAK2V617F-positive Essential Thrombocythemia. Representative immunohistochemical staining for aspartyl-tRNA synthetase (DARS) in bone marrow biopsies (BMB) from patients with essential thrombocythemia (ET). Images show different intensities of cytoplasmic and membranous DARS positivity in clustered megakaryocytes. The staining intensity score in A is + 3, in B and C is + 2, and in D is + 1. Each panel includes a scale bar representing 500 μm. Original magnification 400×

**Fig. 3:**
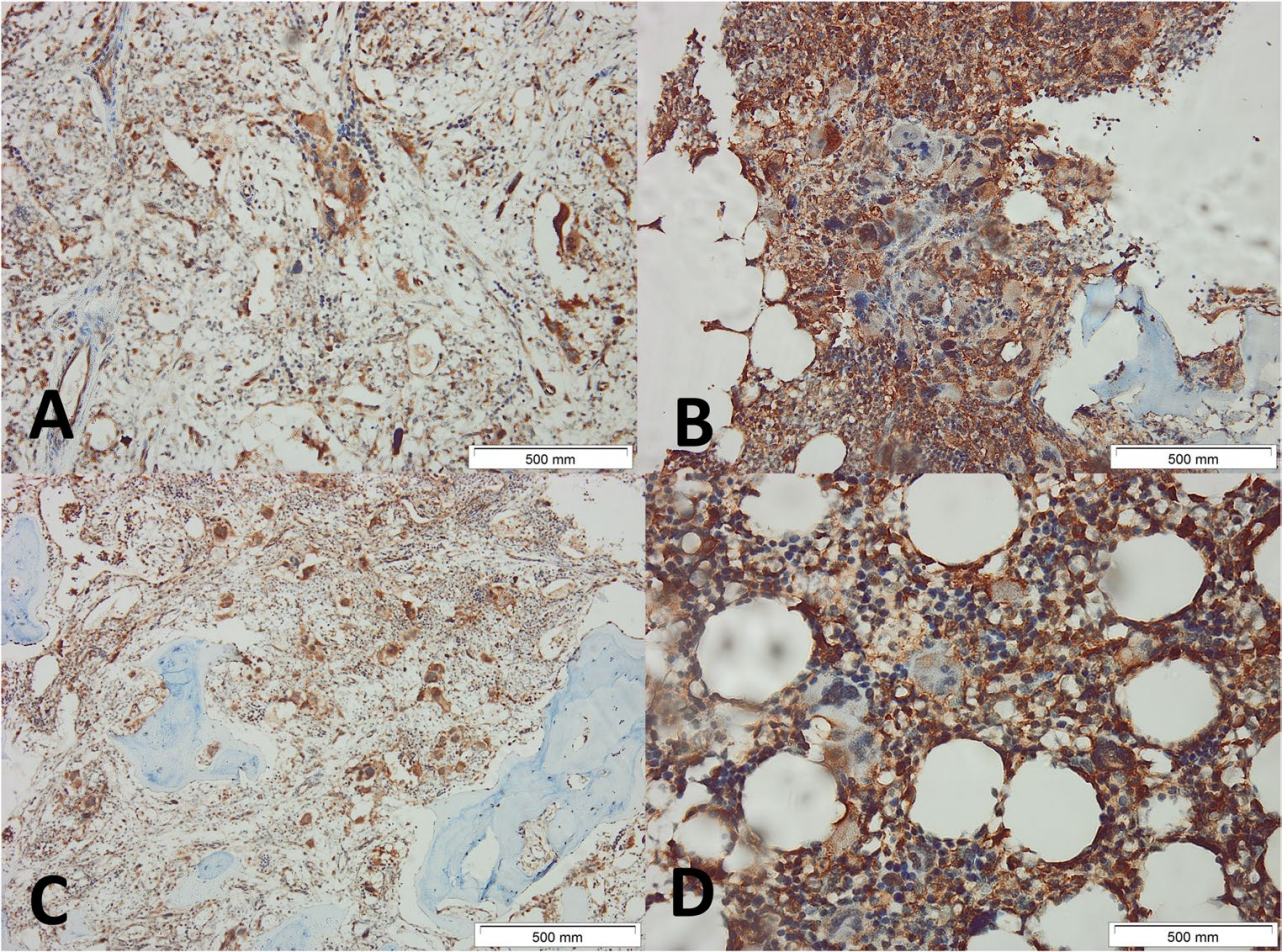
Immunohistochemical Expression of DARS in Bone Marrow Biopsies from Patients with JAK2V617F-positive Primary Myelofibrosis. Representative immunohistochemical staining for aspartyl-tRNA synthetase (DARS) in bone marrow biopsies (BMB) from patients with primary myelofibrosis (PMF). Images A–D illustrate patchy to diffuse cytoplasmic DARS expression in megakaryocytes and residual hematopoietic elements. The fibrotic background and variable cellularity are notable in PMF specimens. Each panel includes a 500 μm scale bar. Original magnification at 100x for A-C and 400× for D

Two experienced hematopathologists (AA and SM) blinded to the clinical data, independently scored DARS intensity (0–3) and percentage of positive cells (0:<1%;1:1–10%;2:11–50%;3:51–80%;4:81–100%) to calculate Immunoreactive score (IRS) (intensity × percentage; 0–12). Discordant cases (< 10%) were reviewed jointly to reach consensus, and another two pathologists (HG and SY) reviewed 20% of cases. Inter-observer κ was 0.89 (95% CI 0.83–0.95). Figures 1, 2 and 3 show examples of different MPNs with different IRSs.

### High vs. low expressor cutoff determination

The cohort median IRS of 9 was prespecified as the biomarker cutoff per REMARK recommendations to avoid data-driven bias [25]. DARS expression cutoff determination was performed using the cohort median (IRS = 9) rather than the previously published cutoff of 6 by Xiong et al. [17]. The latter did not yield adequate group balance or statistical power in our population. Median-based cutoffs are widely validated in biomarker research for maximizing statistical power and ensuring population-specific optimization [25, 26]. This approach accounts for potential differences in patient populations, laboratory conditions, and scoring methodologies between studies.

### Clinical data collection

Clinical parameters included age at diagnosis, gender, constitutional symptoms (B symptoms including fever > 38 °C, night sweats, > 10% unintentional weight loss over 6 months, and loss of appetite), spleen size (measured by abdominal ultrasonography as the maximum dimension in centimeters), and liver status. Laboratory data included complete blood count, lactate dehydrogenase (LDH) levels, JAK2V617F allele burden (quantitative real-time PCR), and bone marrow morphology, including fibrosis grading according to WHO criteria [23]. Risk stratification was performed using established scoring systems (DIPSS for PMF, IPSET-thrombosis for ET, and age-based thrombosis risk stratification for PV).

Survival endpoints included overall survival (OS), defined as time from diagnosis to death from any cause; progression-free survival (PFS), defined as time from diagnosis to disease progression to marrow fibrosis ≥ WHO grade 2 reticulin fibrosis; leukemia-free survival (LFS), defined as time from diagnosis to leukemic transformation; and thrombosis-free survival (TFS), defined as time from diagnosis to first thrombotic event. Follow-up data were collected through medical records and patient contact, with a median follow-up of 37 months (range: 12–84 months).

## Results

### Patient characteristics

Patients were predominantly female (59.5%), with a median age of 59 years (IQR 48–67). PMF was most common (44.6%; *n* = 54), followed by PV (28.1%; *n* = 34), ET (20.7%; *n* = 25), and MPN-U (6.6%; *n* = 8). Median age was highest in MPN-U [65.5 (57.5–70)] and PMF [62 (51.2–68.8)], lowest in ET [55 (49–60)], and intermediate in PV [61 (55–65)]. Splenomegaly was most pronounced in MPN-U [18.5 cm (15.8–19.2)] and PMF [17.5 (13–19)], moderate in PV [13 (12–14.8)], and mildest in ET [12 (11.5–13.5)]. JAK2V617F allele burden was highest in ET [50% (40–75)], followed by PV [39% (26.5–65.8)] and PMF [40% (25–55)], and lowest in MPN-U [24.5% (20.5–61.8)].

### DARS expression patterns across MPN subtypes

**Fig. 4:**
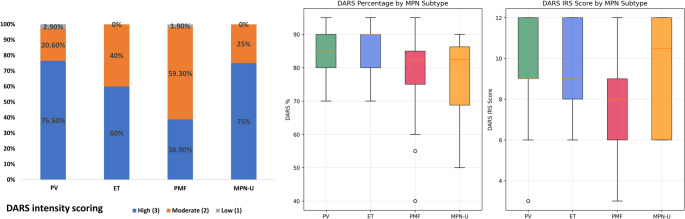
DARS expression characteristics across myeloproliferative neoplasm (MPN) subtypes in BCR::ABL1-negative, JAK2V617F-positive patients (A) Stacked bar chart demonstrates the distribution of DARS intensity scores—high (blue), moderate (orange), and low (gray)—across diagnostic groups: essential thrombocythemia (ET), MPN-unclassifiable (MPN-U), primary myelofibrosis (PMF), and polycythemia vera (PV) (B) Boxplots depict the quantitative assessment of DARS, including DARS percentage (left) and DARS IRS score (right), according to MPN subtype. These analyses reveal distinct patterns and heterogeneity of DARS expression among the different MPN clinical categories, with notable trends toward higher intensity and IRS scores in PV and MPN-U compared to PMF

DARS expression was detected in all examined cases, with significant heterogeneity across MPN subtypes. Subtype-specific analysis revealed distinct expression patterns: PV patients demonstrated the highest DARS expression (mean intensity, percentage of positive cells, and IRS score). ET patients showed comparable levels. PMF patients exhibited significantly lower expression levels in terms of intensity, percentage of positive cells, and IRS, while MPN-U patients showed intermediate levels (Table 1; Fig. 4).

**Table 1:** Summary of DARS Expression Metrics Across MPN Subtypes

Group	Mode Intensity	Mode Count (%)	Intensity Distribution (0/1/2/3, *n*%)	DARS % Median (IQR)	DARS IRS Median (IQR)
PV (*n* = 34)	Strong	26 (76.5)	0:0 (0); 1:1 (2.9); 2:7 (20.6); 3:26 (76.5)	85 (80–90)	9 (9–12)
ET(*n* = 25)	Strong	15 (60)	0:0 (0); 1:0 (0); 2:10 (40); 3:15 (60)	90 (80–90)	9 (8–12)
PMF (combined) (*n* = 54)	Moderate	32 (59.3)	0:0 (0); 1:1 (1.9); 2:32 (59.3); 3:21 (38.9)	82.5 (75–85)	8 (6–9)
Prefibrotic PMF (*n* = 16)	Strong	10 (62.5)	0:0 (0); 1:0 (0); 2:6 (37.5); 3:10 (62.5)	85 (80–86.2)	9 (8–12)
Fibrotic PMF (*n* = 38)	Moderate	26 (68.4)	0:0 (0); 1:1 (2.6); 2:26 (68.4); 3:11 (28.9)	80 (75–85)	8 (6–9)
MPN-U (*n* = 8)	Strong	6 (75)	0:0 (0); 1:0 (0); 2:2 (25); 3:6 (75)	82.5 (68.8–86.2)	10.5 (6–12)

Intensity categories: 0 = negative, 1 = weak, 2 = moderate, 3 = strong

DARS % and IRS scores are non-normal; medians and IQRs are reported

*PV* polycythemia vera, *ET* essential thrombocythemia, *PMF* primary myelofibrosis, *MPN-U* myeloproliferative neoplasm unclassified, *IRS* immunoreactive score, *DARS* Aspartyl-tRNA synthetase

Kruskal-Wallis testing revealed significant differences in DARS expression across MPN subtypes for all parameters: intensity, percentage, and IRS score. Post-hoc pairwise comparisons with Bonferroni correction identified significant differences between PV and PMF for both DARS intensity and IRS score, indicating medium to large effect sizes. No other pairwise comparisons reached statistical significance after correction for multiple testing (all *p* > 0.0083) (Table 2).

**Table 2:** Kruskal-Wallis Tests and Significant Pairwise Comparisons for DARS Expression

DARS Parameter	Kruskal-Wallis H	*p* Value	η²	Post-Hoc PV vs. PMF (Bonferroni-corrected)
Intensity	12.743	0.0052	0.083	*p* = 0.0011 Cohen’s *r* = 0.348 95% CI: 0.15–0.59
Percentage	8.545	0.0360	0.047	No pairwise differences remained significant (*p* > 0.0083)
IRS Score	14.186	0.0027	0.096	*p* = 0.0004 Cohen’s *r* = 0.381 95% CI: 0.81–2.49

**η²**: Eta-squared effect size

Post-hoc tests used Mann-Whitney U with Bonferroni correction (α = 0.0083)

*DARS* Aspartyl-tRNA synthetase, *IRS* immunoreactive score

### Comparison of DARS Expression Groups

Clinical and pathological characteristics differed significantly between low and high DARS expression groups (Table 3).

**Table 3:** Association of Clinical and Pathological Characteristics by DARS IRS Score Groups

Variable	Low DARS IRS Score (< 9)	High DARS IRS Score (≥ 9)	*P* value
	Count (%)	Count (%)	
Diagnosis			0.002
PV	8 (14.5)	26 (39.4)	
ET	10 (18.2)	15 (22.7)	
PMF	34 (61.8)	20 (30.3)	
MPN-U	3 (5.5)	5 (7.6)	
Subclassification of PMF			0.058
Prefibrotic phase	7 (20.6)	9 (45)	
Fibrotic phase	27 (79.4)	11 (55)	
Gender			0.919
Male	22 (40)	27 (40.9)	
Female	33 (60)	39 (59.1)	
Constitutional symptoms*	21 (38.2)	17 (25.8)	0.143
Liver status			0.221
Normal	30 (54.5)	46 (69.7)	
Hepatomegaly	9 (16.4)	8 (12.1)	
Cirrhosis	16 (29.1)	12 (18.2)	
HCV positive	13 (23.6)	5 (7.6)	0.013
Microvessel density			0.602
Low	14 (37.8)	10 (40)	
Moderate	19 (51.4)	10 (40)	
High	4 (10.8)	5 (20)	
WHO Reticulin fibrosis grade			0.006
0	13 (23.6)	23 (34.8)	
1	9 (16.4)	21 (31.8)	
2	8 (14.5)	11 (16.7)	
3	25 (45.5)	11 (16.7)	
WHO Collagen grade (Masson trichrome stain)			0.024
0	22 (40)	43 (65.2)	
1	14 (25.5)	13 (19.7)	
2	11 (20)	4 (6.1)	
3	8 (14.5)	6 (9.1)	
IPSET-Thrombosis (for ET and prefibrotic PMF)			0.425
Low	17 (38.6)	18 (47.4)	
High	27 (61.4)	20 (52.6)	
DIPSS			0.62
0	0 (0)	0 (0)	
1	6 (17.6)	4 (20)	
2	5 (14.7)	5 (25)	
3	10 (29.4)	3 (15)	
4	9 (26.5)	4 (20)	
5	4 (11.8)	4 (20)	
Age-based risk stratification (for PV)			1
Low	4 (50)	11 (42.3)	
High	4 (50)	15 (57.7)	
Outcomes			
Thrombosis Events, n(%)	22 (40)	20 (30.3)	0.26
Leukemic Transformation, n(%)	13 (23.6)	4 (6.1)	0.006
Progression to Fibrosis (*n* = 59), n(%) **	4/18 (22.2)	8/41 (19.5)	0.81
Deaths, n(%)	19 (34.5)	15 (22.7)	0.15

*Constitutional symptoms included fever more than 38 °C and or loss of 10% of body weight in less than 6 months

**Progression to fibrosis was assessed in polycythemia vera and essential thrombocythemia

*PV* polycythemia vera, *ET* essential thrombocythemia, *PMF* primary myelofibrosis, *MPN-U* MPN unclassifiable, *IRS* immunoreactive score, *IPSET* International Prognostic Score of Thrombosis for ET, *DIPSS* Dynamic International Prognostic Scoring System

### DARS expression and clinical parameter correlations

**Fig. 5:**
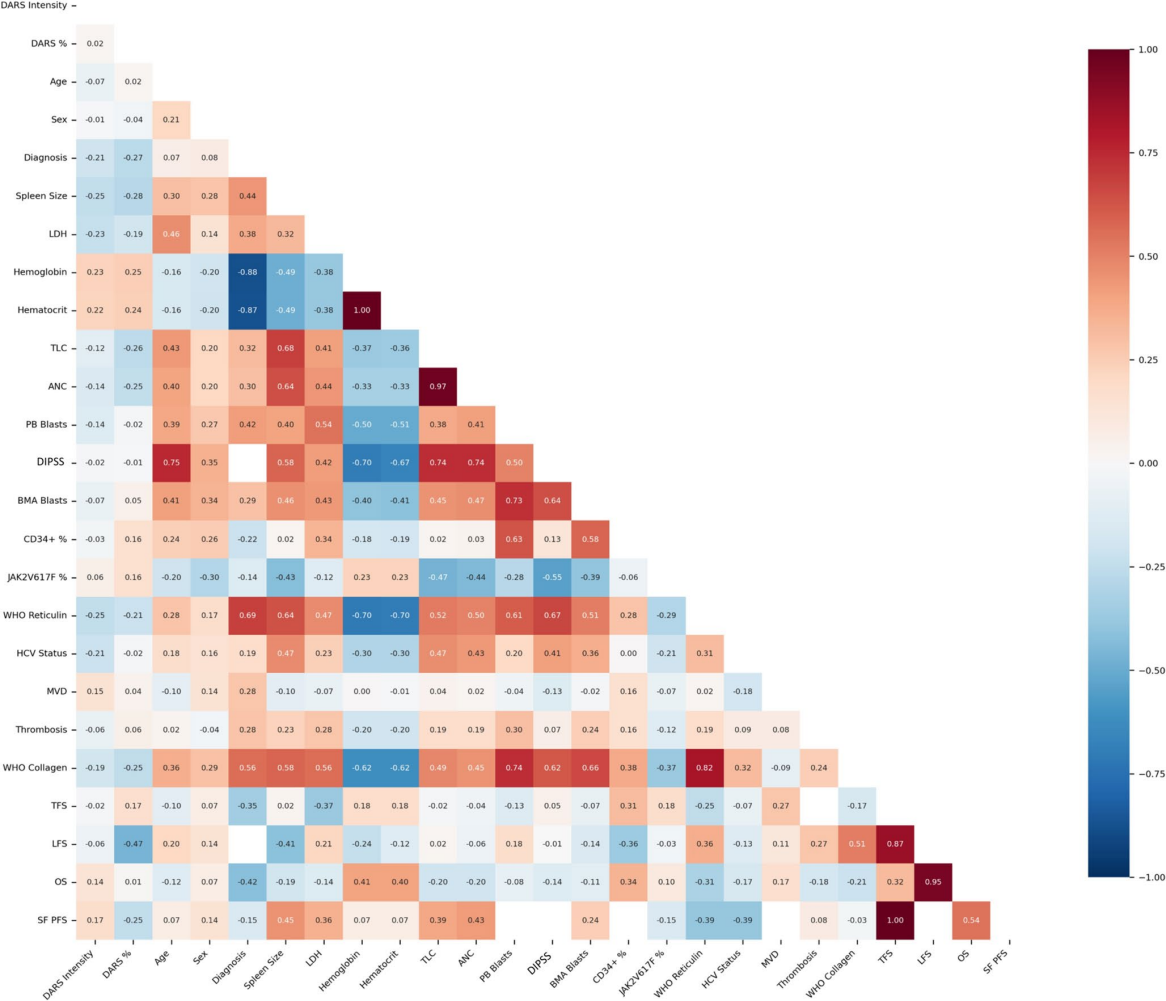
Correlation matrix illustrating pairwise relationships among clinical, laboratory, and molecular parameters in patients with BCR::ABL1-negative, JAK2V617F-positive myeloproliferative neoplasms. The matrix highlights correlations between DARS metrics (intensity, percentage of positive cells in bone marrow, and immunoreactive score (IRS)) and other key variables. Abbreviations: LDH, lactate dehydrogenase; TLC, total leucocytic count; ANC, absolute neutrophilic count; PB, peripheral blood; DIPSS, dynamic international prognostic scoring system; BMA, bone marrow aspirate; WHO, World Health Organization; MVD, microvessel density; TFS, thrombosis-free survival; LFS, leukemia-free survival; OS, overall survival; SF-PFS, secondary fibrosis progression-free survival

Comprehensive correlation analysis revealed significant associations between DARS expression and multiple clinical parameters. DARS IRS score showed significant negative correlations with spleen size, LDH levels, total leukocyte count, and WHO reticulin fibrosis grade. Positive correlations were observed between DARS IRS score and hemoglobin levels, hematocrit, and CD34-positive cells percentage. Similar correlation patterns were observed for DARS intensity and percentage scores (Supplementary Table 1, Fig. 5).

Recognizing hemoglobin’s differential significance (erythrocytosis in PV vs. anemia in PMF), we performed Spearman correlations between DARS metrics and spleen size/hemoglobin, stratified by subtype (Table 4, Supplementary Fig. 1).

**Table 4:** Spearman correlation coefficients (ρ) between DARS metrics and clinical parameters (spleen size, hemoglobin) stratified by MPN subtype

Subtype (*n*)	Metric vs. Spleen Size	ρ	*P*-value	Metric vs. Hemoglobin	ρ	*P*-value
PV (34)	IRS	0.044	0.803	IRS	0.061	0.730
	Intensity	0.135	0.448	Intensity	0.136	0.444
	%	−0.185	0.295	%	0.082	0.647
ET (25)	IRS	0.053	0.800	IRS	−0.346	0.090
	Intensity	0.177	0.397	Intensity	−0.363	0.075
	%	−0.210	0.315	%	−0.010	0.962
PMF (54)	IRS	−0.248	0.070	IRS	0.204	0.139
	Intensity	−0.243	0.077	Intensity	0.029	0.837
	%	−0.099	0.479	%	0.287	0.036

ρ = Spearman's rank correlation coefficient.

*PV* polycythemia vera, *ET* essential thrombocythemia, *PMF* primary myelofibrosis

### Survival analyses

**Fig. 6:**
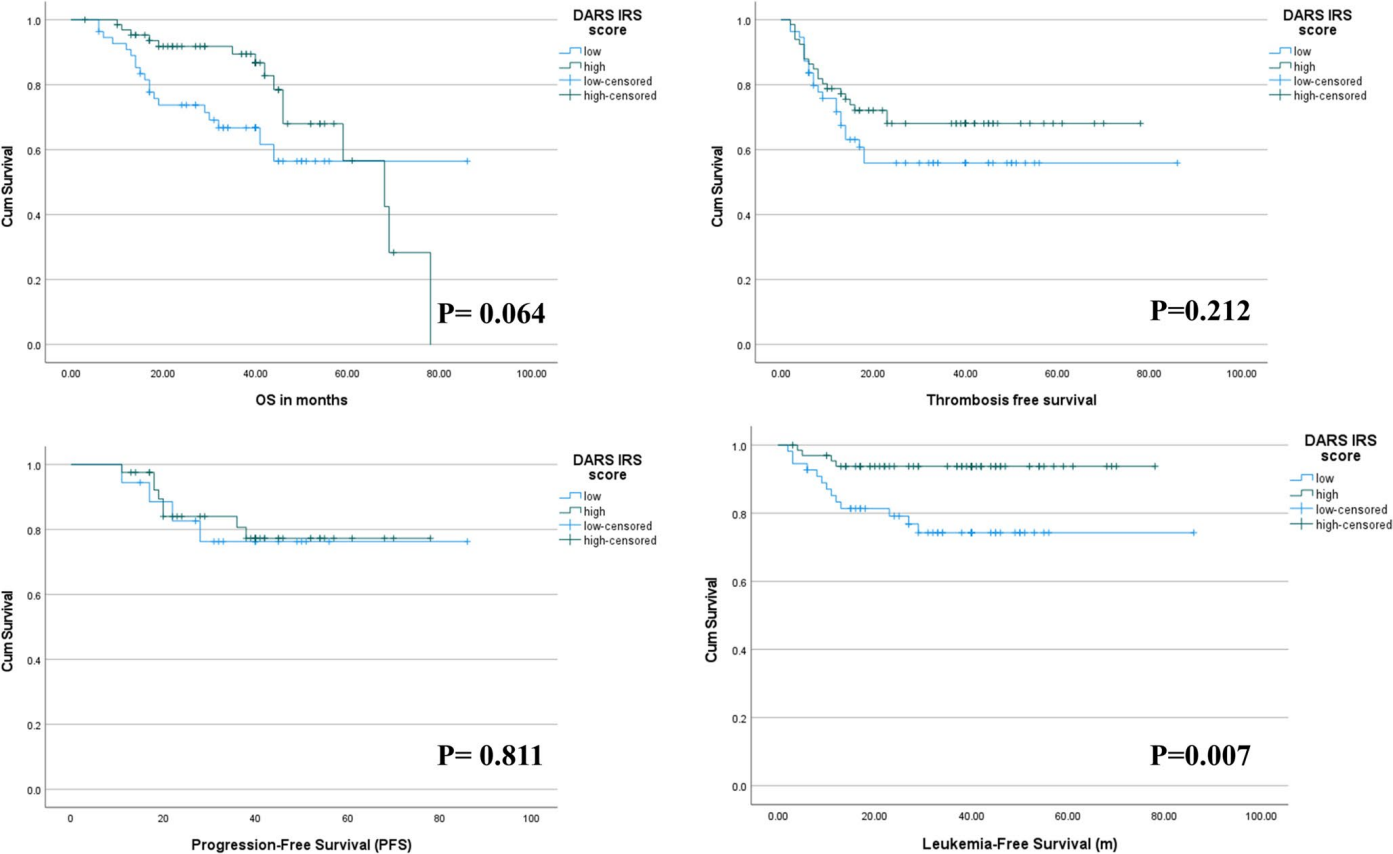
Kaplan-Meier survival curves for patients with BCR::ABL1-negative, JAK2V617F-positive myeloproliferative neoplasms (MPNs) stratified by DARS IRS score (high vs. low). Analyses include overall survival (OS), thrombosis-free survival, secondary fibrosis progression-free survival (PFS) in both PV and ET, and leukemia-free survival (LFS), each shown in months. The log-rank p-values for each comparison are displayed on the corresponding plots; a significant difference in LFS was observed between groups (*P* = 0.007), whereas differences in OS (*P* = 0.064), thrombosis-free survival (*P* = 0.212), and PFS (*P* = 0.811) were not statistically significant

During a median follow-up of 37 months (IQR: 24–52 months), survival data were available for all 121 patients. Patients were stratified by DARS IRS score using the median value (IRS = 9) as the cutoff. Kaplan-Meier analysis revealed significant differences in LFS between high and low DARS expression groups. Patients with high DARS expression demonstrated superior LFS with a median not reached versus 67.2 months in the low expression group. Cox proportional hazards analysis identified a high DARS IRS score as an independent predictor of improved LFS (HR = 0.42, *p* = 0.007, 95% CI: 0.22–0.80) after adjusting for age, MPN subtype, spleen size, and WHO fibrosis grade. No significant associations were observed for OS (*p* = 0.064), secondary fibrosis PFS (*p* = 0.811), or TFS (*p* = 0.212) (Supplementary Table 2, Fig. 6).1

## Discussion

 This comprehensive study represents the largest cohort analysis of DARS expression in BCR::ABL1-negative JAK2V617F-positive MPNs to date, providing insights into its differential expression patterns across MPN subtypes and significant associations with clinical parameters and survival outcomes. Our findings substantially extend the pioneering work of Xiong et al. [17] by providing the first comprehensive statistical analysis of DARS heterogeneity across MPN subtypes.

### DARS overexpression and differential expression across MPN subtypes

Our results confirm and extend the fundamental observation that DARS is significantly overexpressed in all MPN subtypes. However, our study provides the first comprehensive statistical analysis demonstrating significant heterogeneity in DARS expression across different MPN subtypes, with PV showing the highest expression levels compared to PMF. This hierarchical expression pattern (PV > ET > MPN-U > prefibrotic PMF > fibrotic PMF) may reflect underlying biological differences between these entities and suggests potential diagnostic utility. The observed differential expression aligns with recent findings in aminoacyl-tRNA synthetase biology, where these enzymes have been recognized as key regulators of cellular metabolism and stress responses [27, 28]. The higher DARS expression in PV, characterized by erythroid proliferation and typically associated with the highest JAK2V617F allele burden, suggests that DARS upregulation may be linked to proliferative capacity and metabolic demands of the malignant clone. While no studies have yet characterized DARS expression in blast-phase MPN (MPN-BP) or secondary AML post-MPN, our data predict downregulation in leukemic transformation. We observed a progressive decline in DARS across disease states, with fibrotic PMF (closest to blast phase) showing the lowest DARS expression. High DARS predicted superior LFS, positioning DARS loss as a potential marker of leukemogenic progression, warranting future blast-phase validation.

### Mechanistic insights and pathophysiological implications in PMF

The significantly lower DARS expression in PMF, particularly in fibrotic versus prefibrotic cases (*p* = 0.0208 for intensity, *p* = 0.0223 for IRS), provides new insights into PMF pathogenesis. Recent studies have demonstrated that mitochondrial dysfunction and altered protein synthesis are features of MPN progression [29, 30]. As PMF progresses from prefibrotic to fibrotic phases, the reduction in DARS expression may reflect decreased cellular metabolic activity, increased apoptosis, or alterations in the bone marrow microenvironment associated with fibrosis development. Furthermore, DARS2 has been shown to orchestrate iron-sulfur metabolism in hematopoietic stem cells through alternative RNA splicing mechanisms, suggesting that DARS family members may have specialized roles in normal and malignant hematopoiesis [31].

### Spleen and hemoglobin insights

 In the overall cohort, the strong inverse correlation between DARS expression and spleen size (*r* = -0.266, *p* = 0.003) represents a particularly intriguing finding. This relationship suggests that patients with higher DARS expression may have less advanced or better-controlled disease, as splenomegaly is typically associated with disease progression and burden in MPNs [32]. Similarly, the positive correlation between DARS expression and hemoglobin levels (*r* = 0.308, *p* = 0.001) supports this hypothesis, as higher hemoglobin levels are generally associated with better disease control and patient outcomes. While overall cohort analysis revealed significant correlations between DARS and spleen size/hemoglobin, MPN subtype-stratified analysis (Table 4) demonstrated that these relationships were primarily driven by between-subtype differences rather than within-subtype associations. The hierarchical DARS expression pattern (PV > ET> PMF) coincides with characteristic clinical phenotypes (small spleens/erythrocytosis in PV vs. large spleens/anemia in PMF), creating the appearance of correlation when subtypes are pooled. This finding underscores the importance of subtype-specific biomarker validation in MPN research.

#### Integration with tumor microenvironment biology

The inverse correlation between DARS expression and fibrosis grade (ρ=-0.280, *p* = 0.002) parallels recent findings by Xiong et al. [33] demonstrating that DARS promotes M2 polarization of tumor-associated macrophages (TAMs) in MPNs. Their mechanistic studies revealed that DARS knockdown in macrophages reduced IL-6, IL-8, IL-10, and IFN-α secretion, decreased M2-polarized cells, and inhibited MPN cell proliferation. This bidirectional relationship—where DARS expression in both malignant cells and macrophages drives M2 polarization—creates a feed-forward loop promoting disease progression. Our observation of lower DARS in advanced PMF may reflect the exhaustion of this protumorigenic axis as the microenvironment transitions toward a fibrosis-dominated pathology.

#### Fibrosis as a terminal microenvironmental state

The progressive decline in DARS expression from PV to prefibrotic PMF to fibrotic PMF (*p* = 0.001) aligns with the established model of bone marrow fibrosis as a reactive, non-clonal process driven by aberrant megakaryocyte-stromal cell interactions. Studies have shown that collagen deposition correlates with disease severity and represents an advanced microenvironmental alteration that may ultimately constrain metabolically active hematopoiesis. Our data suggest that DARS downregulation accompanies this transition, potentially due to reduced cellular density, altered stromal signaling, or metabolic reprogramming in the fibrotic niche. This interpretation is consistent with transcriptomic studies showing downregulation of protein synthesis machinery in advanced myelofibrosis.

### Prognostic significance and survival implications

Our survival analysis revealed that high DARS expression is associated with improved LFS (HR = 0.42, *p* = 0.007). This finding suggests that DARS expression levels may serve as a predictor of leukemic transformation risk. The lack of association with OS may reflect the relatively short follow-up period and the indolent nature of many MPNs. These findings are consistent with recent studies demonstrating the prognostic value of metabolic biomarkers in hematological malignancies [34, 35]. The role of aminoacyl-tRNA synthetases in maintaining protein homeostasis and cellular stress responses positions DARS as a potential guardian against malignant transformation, explaining its positive prognostic implications.

#### Mechanistic basis for DARS as a protective factor

The protective effect of high DARS expression on leukemic transformation (HR = 0.42) appears paradoxical, given Xiong et al.‘s [33] findings that DARS promotes MPN cell proliferation and M2 macrophage polarization. We propose several non-mutually exclusive explanations: (1) Metabolic fidelity hypothesis—High DARS maintains translational accuracy and proteostasis, preventing accumulation of additional mutations required for blast transformation. Studies in myelodysplastic syndromes have identified altered cytoplasmic tRNA synthetase levels as therapeutic vulnerabilities, suggesting these enzymes safeguard against genomic instability. (2) Threshold effect model—DARS may promote early MPN proliferation but protect against transformation, similar to the JAK2V617F allele burden’s relationship with phenotype. (3) Microenvironmental exhaustion—Progressive DARS loss reflects microenvironmental deterioration (fibrosis, vascular remodeling) that creates a permissive niche for blast expansion. (4) Clonal evolution—Leukemic subclones may downregulate DARS as part of metabolic reprogramming toward glycolytic metabolism, a hallmark of acute leukemias. Validation studies comparing paired chronic-phase and blast-phase samples are needed to distinguish whether DARS loss is causative or consequential in transformation.

#### **Comparative analysis with Xiong et al.** [17] f**indings**

While both studies demonstrate DARS overexpression in MPNs, a key difference emerges: Xiong et al. reported associations between high DARS and disease burden (splenomegaly, elevated LDH), whereas we found inverse correlations in the whole cohort (high DARS with lower spleen size, lower LDH, higher hemoglobin). This discrepancy may reflect: (a) Different cutoff methodologies (IRS = 6 vs. median-based IRS = 9), (b) Cohort heterogeneity (our JAK2V617F-exclusive cohort vs. their broader MPN population), or (c) Nonlinear dose-response relationships where moderate DARS elevation drives pathology but extreme elevation reflects compensatory metabolic stress.

### Clinical translation and biomarker potential

The differential DARS expression patterns suggest several potential clinical applications. First, as a diagnostic aid, the expression hierarchy could assist in classifying challenging cases, particularly in distinguishing prefibrotic PMF from ET or early PV. Second, as a candidate prognostic marker, DARS expression might guide treatment intensity and monitoring frequency. Third, as a potential therapeutic target, the overexpression across all subtypes suggests that DARS-targeted therapies might have broad applicability in MPNs. The excellent inter-observer agreement in DARS scoring supports its potential for routine clinical use.

#### Implementation pathway for clinical adoption

For DARS to transition from research biomarker to clinical tool, several milestones must be achieved: (1) Standardization: Establishment of standardized IHC protocols (antibody clone, dilution, scoring system) with multi-institutional validation. The IRS scoring system used in our study and Xiong et al.‘s [17] work provides a reproducible framework but requires inter-laboratory calibration. (2) Threshold validation: Our median-based cutoff (IRS = 9) requires validation in independent cohorts with diverse demographics. Receiver operating characteristic analysis in larger cohorts could optimize cutoffs for specific clinical questions (e.g., predicting transformation vs. thrombosis). (3) Integration with existing scores: DARS expression should be tested as an adjunct to established risk scores (DIPSS for PMF, IPSET-thrombosis for ET). Composite models incorporating DARS, genetic mutations (e.g., ASXL1, SRSF2), and clinical parameters may improve prognostic accuracy. (4) Prospective validation: Retrospective findings must be confirmed in prospective cohorts with pre-specified endpoints and standardized follow-up protocols.

### Therapeutic implications

The role of DARS in protein synthesis and cellular metabolism positions it as an attractive therapeutic target. Recent studies have explored aminoacyl-tRNA synthetase inhibitors as anti-cancer agents. The differential expression patterns we observed suggest that DARS-targeted therapies may have varying efficacy across MPNs, with potentially greater benefit in high-expressing subtypes such as PV. Moreover, recent evidence demonstrates that leucyl-tRNA synthetase promotes malignant progression in hematological malignancies by regulating the glycolytic pathway, supporting the potential for targeting aminoacyl-tRNA synthetases in blood cancers.

### Limitations

Several limitations should be acknowledged. First, our multicenter cohort revealed an unexpected median JAK2V617F allele burden in ET, exceeding that in PV and PMF. This contrasts with population-based studies, which typically report lower ET burdens (< 30%). Two non-mutually exclusive explanations may underlie this observation: biological heterogeneity of ET, including a subset with genuinely high JAK2V617F load. High ET allele burdens (> 50%) represent legitimate WHO-defined ET, with established literature documenting that 5% of ET cases reach 87% burden [36] and that ≥ 58% thresholds are established [37]. The second observation is that our biopsy-based design inherently selected more proliferative high IPSET-thrombosis risk cases. Tertiary referral patterns across the three university hospitals were enriched for symptomatic ET (40% high IPSET-thrombosis risk vs. 0% constitutional symptoms distinguishing from prefibrotic PMF). Results should be validated in a larger ET cohort that represents both the classic presentation and the high-risk disease. Second, given the retrospective design and the moderate sample size (*n* = 121), these associations require prospective confirmation. Third, the relatively short median follow-up (37 months) may not capture long-term survival differences in these indolent diseases. Fourth, functional studies are needed to establish causal relationships between DARS expression and MPN pathogenesis. Fifth, our study focused exclusively on JAK2V617F-positive cases, limiting generalizability to CALR- or MPL-mutated MPNs. Sixth, we did not evaluate serum DARS protein levels or enzymatic activity, which might provide additional insights into its functional significance. The immunohistochemical approach, while clinically relevant, provides semi-quantitative rather than absolute DARS levels. The correlation between tissue DARS expression and circulating DARS levels, which might be more practical for monitoring, remains unexplored. Finally, Subtype-stratified correlation analyses were limited by modest sample sizes, which provided insufficient power (~ 40–50%) to detect moderate within-subtype correlations. The near-significant trends observed suggest that true associations may emerge in larger cohorts.

## Conclusions

This extensive DARS expression investigation in BCR::ABL1-negative JAK2V617F-positive MPNs shows several critical findings. First, PV has considerably greater DARS levels than PMF, suggesting diagnostic potential. Second, high DARS expression was associated with improved LFS (HR = 0.42, *p* = 0.007), suggesting it as a putative independent risk stratification factor that may inform follow-up tactics in future studies. These findings suggest DARS’s potential utility as a biomarker in MPN classification and prognosis, though validation in larger prospective cohorts and mechanistic studies is needed.

## Supplementary Information

Below is the link to the electronic supplementary material.

Supplementary Material 1 (DOCX 16.5 KB)

Supplementary Material 2 (JPG 2.43 MB)

Supplementary Material 3 (DOCX 14.7 KB)

## Data Availability

The datasets used and analyzed in the current study are available from the corresponding author upon reasonable request, subject to appropriate ethics approval.

## Abbreviations

DARS: Aspartyl-tRNA synthetase
DIPSS: Dynamic International Prognostic Scoring System
ET: Essential thrombocythemia
IPSET: International Prognostic Score for ET
IRS: Immunoreactive score
LDH: Lactate dehydrogenase
LFS: Leukemia-free survival
MPN: Myeloproliferative neoplasm
MPN-U: MPN-unclassifiable
PMF: Primary myelofibrosis
PV: Polycythemia vera
