# Supplementary material for: DARS expression in JAK2V617F-positive myeloproliferative neoplasms: immunohistochemical analysis and clinical associations
Source: Ann Hematol. 2026 Mar 27;105(4):209. doi: 10.1007/s00277-026-06934-0 (PMC13021852; doi:10.1007/s00277-026-06934-0)
Supplement: Supplementary file 1 — Supplementary Material 1 (DOCX 16.5 KB) [file 277_2026_6934_MOESM1_ESM.docx]

Supplementary Table 1 Correlations Between Clinical/Laboratory Parameters and DARS Indices

| Variable | IRS Score (r, P) | DARS Intensity (r, P) | DARS % of positive cells (r, P) |
| --- | --- | --- | --- |
| Age | -0.020, 0.831 | -0.052, 0.569 | -0.071, 0.441 |
| Spleen size (cm) | -0.266, 0.003 | -0.174, 0.056 | -0.279, 0.002 |
| LDH (mg/dl) | -0.194, 0.033 | -0.198, 0.029 | -0.158, 0.083 |
| Hb (g/dl) | 0.308, 0.001 | 0.236, 0.009 | 0.220, 0.015 |
| Hematocrit (%) | 0.296, 0.001 | 0.226, 0.013 | 0.213, 0.019 |
| TLC (×10³/cmm) | -0.234, 0.010 | -0.140, 0.127 | -0.228, 0.012 |
| Absolute neutrophil count | -0.242, 0.008 | -0.152, 0.096 | -0.234, 0.010 |
| Platelets count | -0.013, 0.888 | 0.000, 0.997 | 0.105, 0.251 |
| Pb blasts (%) at diagnosis | -0.127, 0.165 | -0.152, 0.097 | -0.055, 0.550 |
| DIPSS (for PMF) | -0.132, 0.342 | -0.038, 0.784 | -0.088, 0.525 |
| IPSET (for ET) | 0.04, 0.848 | 0.0001, 1 | 0.106, 0.614 |
| Age-based risk stratification (for PV) | 0.052, 0.769 | 0.049, 0.783 | 0.031, 0.86 |
| BMA blasts at diagnosis (%) | -0.017, 0.856 | -0.056, 0.540 | 0.076, 0.405 |
| CD34 positive cells (%) | 0.243, 0.057 | 0.011, 0.933 | 0.280, 0.028 |
| JAK2 V617F (%) | 0.118, 0.196 | 0.042, 0.648 | 0.180, 0.048 |
| WHO Reticulin fibrosis grade | -0.280, 0.002 | -0.247, 0.006 | -0.205, 0.024 |
| WHO Collagen grade (Masson trichrome) | -0.274, 0.002 | -0.221, 0.015 | -0.214, 0.018 |

*Statistically significant (p<0.05). Abbreviations: ρ: Spearman correlation coefficient; CI: confidence interval; IRS: immunoreactive score. Hb: hemoglobin, TLC: total leucocytic count, Pb: peripheral blood, BMA: bone marrow aspirate, CD: cluster of differentiation, WHO: World Health Organization, DIPSS: dynamic international prognostic scoring system, PV: polycythemia vera, ET: essential thrombocythemia, PMF: primary myelofibrosis.
