# Supplementary material for: DARS expression in JAK2V617F-positive myeloproliferative neoplasms: immunohistochemical analysis and clinical associations
Source: Ann Hematol. 2026 Mar 27;105(4):209. doi: 10.1007/s00277-026-06934-0 (PMC13021852; doi:10.1007/s00277-026-06934-0)
Supplement: Supplementary file 3 — Supplementary Material 3 (DOCX 14.7 KB) [file 277_2026_6934_MOESM3_ESM.docx]

Supplementary Table 2 Survival Outcomes by DARS IRS Score Groups

| Survival Outcome | DARS IRS Score | Mean Estimate (95% CI) | P value |
| --- | --- | --- | --- |
| Overall Survival (OS) | Low | 58.5 (48.54 – 68.47) | 0.064 |
|  | High | 59.63 (52.38 – 66.88) |  |
| Fibrosis Progression-Free Survival (PFS)* | Low | 70.3 (56.78 – 83.81) | 0.811 |
|  | High | 65.62 (57.99 – 73.24) |  |
| Leukemia-Free Survival (LFS) | Low | 67.21 (58.32 – 76.10) | 0.007 |
|  | High | 73.63 (69.47 – 77.78) |  |
| Thrombosis-Free Survival (TFS) | Low | 52.33 (41.64 – 63.03) | 0.212 |
|  | High | 56.17 (48.20 – 64.14) |  |

*Fibrosis progression-free survival is measured in polycythemia vera and essential thrombocythemia cases. Abbreviation: CI: confidence interval. DARS aspartyl-tRNA synthetase, IRS: immunoreactive score
